# Deciphering Hierarchical Chromatin Domains and Preference of Genomic Position Forming Boundaries in Single Mouse Embryonic Stem Cells

**DOI:** 10.1002/advs.202205162

**Published:** 2023-01-19

**Authors:** Yusen Ye, Shihua Zhang, Lin Gao, Yuqing Zhu, Jin Zhang

**Affiliations:** ^1^ School of Computer Science and Technology Xidian University Xi'an Shaanxi 710071 P. R. China; ^2^ NCMIS CEMS RCSDS Academy of Mathematics and Systems Science Chinese Academy of Sciences Beijing 100190 P. R. China; ^3^ School of Mathematical Sciences University of Chinese Academy of Sciences Beijing 100049 P. R. China; ^4^ Center for Excellence in Animal Evolution and Genetics Chinese Academy of Sciences Kunming 650223 P. R. China; ^5^ Center for Stem Cell and Translational Medicine School of Life Sciences Anhui University Hefei Anhui 230601 P. R. China; ^6^ Center for Stem Cell and Regenerative Medicine Department of Basic Medical Sciences, and Bone Marrow Transplantation Center of the First Affiliated Hospital Zhejiang University School of Medicine Hangzhou Zhejiang 310003 P. R. China; ^7^ Zhejiang Laboratory for Systems and Precision Medicine Zhejiang University Medical Center Hangzhou Zhejiang 311121 P. R. China; ^8^ Institute of Hematology Zhejiang University Hangzhou Zhejiang 310058 P. R. China; ^9^ Center of Gene/Cell Engineering and Genome Medicine Hangzhou Zhejiang 310058 P. R. China

**Keywords:** chromatin landscape, hierarchical chromatin domains, mouse embryonic stem cells, regulatory factors, retrotransposons, single cell 3D genome

## Abstract

The exploration of single‐cell 3D genome maps reveals that chromatin domains are indeed physical structures presenting in single cells, and domain boundaries vary from cell to cell. However, systematic analysis of the association between regulatory factor binding and elements and the formation of chromatin domains in single cells has not yet emerged. To this end, a hierarchical chromatin domain structure identification algorithm (named as HiCS) is first developed from individual single‐cell Hi‐C maps, with superior performance in both accuracy and efficiency. The results suggest that in addition to the known CTCF‐cohesin complex, Polycomb, TrxG, pluripotent protein families, and other multiple factors also contribute to shaping chromatin domain boundaries in single embryonic stem cells. Different cooperation patterns of these regulatory factors drive genomic position categories with differential preferences forming boundaries, and the most extensive six types of retrotransposons are differentially distributed in these genomic position categories with preferential localization. The above results suggest that these different retrotransposons within genomic regions interplay with regulatory factors navigating the preference of genomic positions forming boundaries, driving the formation of higher‐order chromatin structures, and thus regulating cell functions in single mouse embryonic stem cells.

## Graphical abstract

The authors first developed a hierarchical chromatin domain structure identification algorithm (HiCS) from single‐cell 3D genome maps. Further systematic analysis on single mouse embryonic stem cells suggests that different retrotransposons within genomic regions interplay with regulatory factors navigating the preference of genomic positions forming boundaries, driving the formation of chromatin landscapes, and thus regulating cell functions.

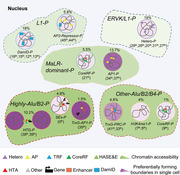

## 1 Introduction

Genome across a wide range of eukaryotic organisms is efficiently packaged and organized into hierarchical chromatin architecture via ubiquitous architectural features, which is critical to gene regulation and dynamical changes in development and disease.^[^ ^1^ ^]^ These basic features consist of chromatin fibers, which fold into chromatin loops, such as enhancer‐promoter interactions and architectural loops mediated by the CCCTC‐binding factor (CTCF).^[^ ^2^ ^]^ These fibers further fold into chromatin domains, referred to as topologically associating domains (TADs) or sub‐TADs, which are associated with each other to generate chromosomal compartments. Each chromosome occupies a distinct volume or chromosome territory within the nucleus.^[^ ^2^, ^3^ ^]^ Genome architecture is an integral part of the chromatin landscape that transcription factors (TFs) must navigate to exert their regulatory roles.^[^ ^4^ ^]^ Although most loci and chromosomes are characterized by a high degree of order and non‐randomness, the precise functional roles and formation mechanisms of these features remain obscure.^[^ ^5^ ^]^

Some TFs, cofactors, and histone modifications that correlate with the chromatin structures have been identified to study features of chromatin organization.^[^ ^6^ ^]^ Particularly, TAD boundaries are enriched with multiple factors including CTCF, cohesin, H3K4me3, H3K36me3, transcription start sites, and housekeeping genes, suggesting that CTCF binding, high levels of transcription activity, multiple histone modifications, and other regulatory factors may contribute to the formation of chromatin domains in mammals.^[^ ^5^ ^]^ Although TADs are highly conserved and stable across different cell types,^[^ ^3^, ^7^ ^]^ single‐cell 3D genome analysis indicated that they display substantial cell‐to‐cell variation.^[^ ^8^ ^]^ Therefore, the bulk analysis only reflects properties of ensemble structures from millions of cells, which may mask chromatin features appearing in a few cells or a single cell.

A recent study reveals that domain structures often adopt globular conformation with spatially physical segregation, and domain boundaries are preferentially located at CTCF‐ and cohesin‐binding sites with a super‐resolution chromatin tracing method.^[^ ^8a^ ^]^ More surprisingly, single‐cell domain structures persist even after cohesin degradation.^[^ ^8a^ ^]^ These results suggest other TFs or epigenetic factors may contribute to the formation of chromatin domains. Currently, mouse embryonic stem cells (mESCs) have served as a specific model to elucidate the mechanisms of 3D genome organization. Hundreds of TFs and epigenetic modification profiles have been identified in mESCs.^[^ ^6^ ^]^ The above observations support us to investigate the formation of chromatin domains and their relationship with functional elements in single mESCs systematically.

Here, we first develop a hierarchical chromatin domain structure identification algorithm (named HiCS) from single‐cell Hi‐C maps, which shows superior performance in both accuracy and efficiency. We reorganize an atlas of ChIP‐seq for mESCs and reveal hundreds of regulatory factors are significantly either enriched or absented in domain boundaries of single cells, suggesting that, in addition to known CTCF‐cohesin complex, Polycomb, TrxG, pluripotent protein families, different types of histone modifications, and other multiple factors could promote the formation of chromatin domains in single mESCs. To further elaborate on genomic position categories with differential preference forming boundaries, we cluster 13 large genomic position categories (consisting of 29 sub‐categories) annotated by 7 different regulatory factor clusters (consisting of 27 sub‐clusters). The clear patterns provide a detailed view of the preference of these genomic position categories forming boundaries. Furthermore, we discover that these genomic position categories are enriched by different cooperation of retrotransposons with preferential localization. Last but not the least, we find that genomic positions enriched by Alu/B2/B4 retrotransposons have higher preference scores for forming boundaries in G1 and ES phases in comparison with MS and LS/G2 phases, whereas genomic positions enriched by L1/ERVK retrotransposons display opposite tendency. In summary, we reveal that multiple types of regulatory factors interplaying with each other in specific genomic positions could affect focal chromatin interactions, thereby changing the interaction density or insulation strength of these regions. This further navigates the preference of genomic position forming boundaries, shapes hierarchical chromatin domains, and thus regulates gene expression and cell functions, even cell identity in single embryonic stem cells.

## 2 Results

### 2.1 Overview of HiCS

**Figure 1:**
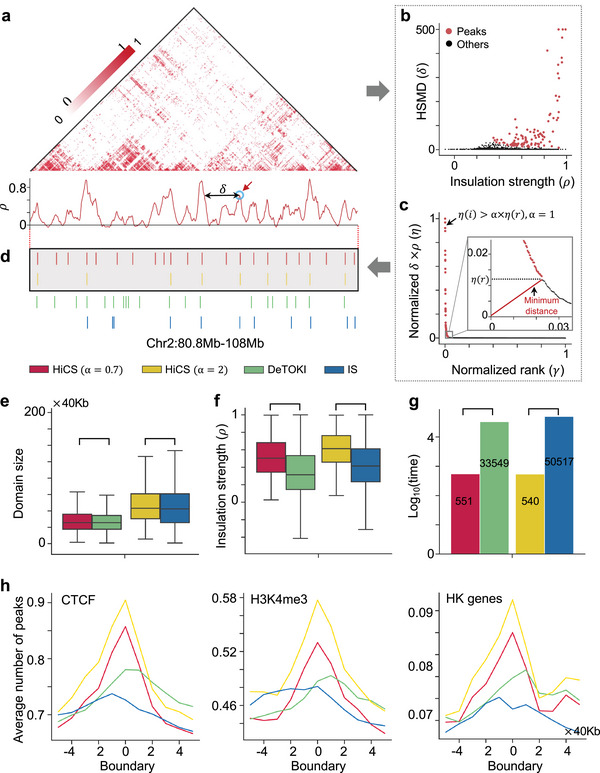
Illustration and efficiency of HiCS for determining the chromatin domains. a) An illustrative example of the preprocessed single‐cell Hi‐C contact map (top) and the insulation strength (*ρ*) of genomic positions (bottom). HSMD (*δ*) represents the minimum distance between the bin and any other bins with higher strengths. b,c) The decision graph (b) and the normalization value of *η* = *ρ* × *δ* in a decreasing order (c) for the domain boundaries (colored in red) in the optimal structural identification parameter. The decision graph of optimal structural identification parameter (zoom box in (c)). d) A local example of domain boundaries from the map in (a) at the region Chr2:80.8—108 Mb with different methods. e,f) Comparison of different methods for domain sizes and insulation strengths. g) Runtime (seconds) of different methods or parameters. h) The average number of CTCF peaks, H3K4me3 peaks, and HK genes at domain boundaries of single cells. The above results (d–h) are implemented by different methods across all single cells.

**Figure 2:**
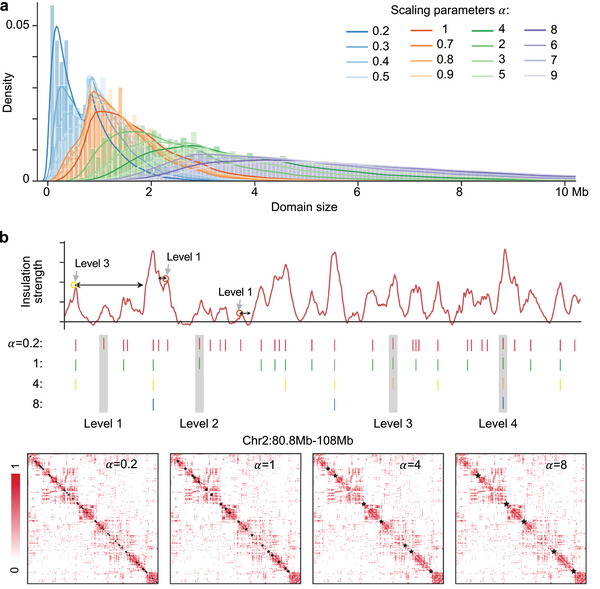
Identification of hierarchical chromatin domains. a) Density distribution of domain sizes at different multi‐scale parameters. b) An example for hierarchical chromatin domains at the region Chr2:80.8–108 Mb. Circles mark three boundaries (two boundaries in level 1 have a magnitude difference in local insulation strength, and the boundary in level 3 has lower insulation strength than one of the boundaries in level 1).

The key design of HiCS is to convert the problem of the identification of hierarchical chromatin domains into finding peaks of insulation strength at different genome scales. The domain boundaries usually have higher insulation strength than their neighbors and a relatively large distance from any regions with higher strength (**Figure** **1**a). HiCS calculates two metrics for each bin including the insulation strength *ρ* and the minimum distance between the bin and any other bin with higher strength *δ*, and controls the number of peaks to obtain the hierarchical chromatin domains by scaling parameter *α* (Figure 1b,c). HiCS is super‐fast to identify a chromatin hierarchy that the domain of a higher level embraces the multiple smaller ones of a lower level (**Figure** **2**b). Note that high‐level boundaries with high *δ* in local regions may have lower insulation strength than low‐level ones (Figure 2b).

Specifically, HiCS consists of three steps (Experimental Section): 1) Preprocesses single‐cell Hi‐C data and calculates the two metrics to find the peaks at a given scale *α*, and determines the domain boundaries for each chromosome of an individual cell (Figure 1a–c); 2) determines the hierarchical domains of chromatin by adjusting the optimal structural identification parameter (*α* = 1) (Figures 1c and 2a,b and Figure S1a, Supporting Information), Noting that the optimal parameter is automatically determined by the algorithm; 3) applies a bi‐clustering method to group chromatin positions and regulatory factors respectively and analyzes the genomic structure‐function relationship by combining hundreds of TFs and epigenetic factors with chromatin domains (Figure 4).

### 2.2 HiCS Shows Superior Performance

We benchmark the performance of HiCS for domain detection against two methods: one is the commonly used method for bulk data, insulation score (IS),^[^ ^9^ ^]^ and another is a recent single‐cell TAD detection method, deTOKI.^[^ ^10^ ^]^ We apply these methods to the preprocessed single‐cell Hi‐C data generated from mESCs.^[^ ^11^ ^]^ To compare their performances fairly, we adjust the scaling parameter of HiCS to obtain similar number and size of domains with IS and deTOKI, respectively (Figure 1e). Actually, the insulation strengths of domains obtained from HiCS are significantly higher than those of IS and deTOKI respectively with similar number of domains, suggesting its superiority to competing methods (Figure 1f). The running time of HiCS is significantly less than both algorithms under the same hardware condition (Figure 1g). Moreover, the domain boundaries detected by HiCS are more significantly enriched in multiple common factors, including CTCF, H3K4me3, Housekeeping (HK) genes (Figure 1h), as well as RNA polymerase II (PolII), promoters, highly expressed genes, and average phastCons score (Figure S1b, Supporting Information). Also, enhancers or super enhancers (SEs) unfavorably form domain boundaries in single cells as reported in the analysis of bulk cells^[^ ^6a^ ^]^ (Figure S1b, Supporting Information). With an example, we can see that HiCS can obtain more accurate chromatin domain boundaries at single‐cell resolution (Figure 1a–d). Taken together, HiCS shows superior performance in both accuracy and efficiency.

### 2.3 The Existence of Hierarchical Chromatin Domains

We adjust the optimal structural identification parameter to generate multiple‐scale chromatin domains at different genomic scales for 1315 single mESCs at 40 kb resolution. We clearly observe four peaks of domain size and insulation strength distribution with different scaling parameters of 0.2, 1, 4, and 8, and choose these scaling parameters for the downstream analysis (Figure 2a,b and Figure S1a, Supporting Information). The domain scales of these four levels are ≈200 to 600 Kb, ≈800 Kb to 1 Mb, ≈2 to 3 Mb, and ≈5 Mb respectively. We show an example of hierarchical chromatin domains, which well match with the local insulation strength of chromatin regions (Figure 2b).

**Figure 3:**
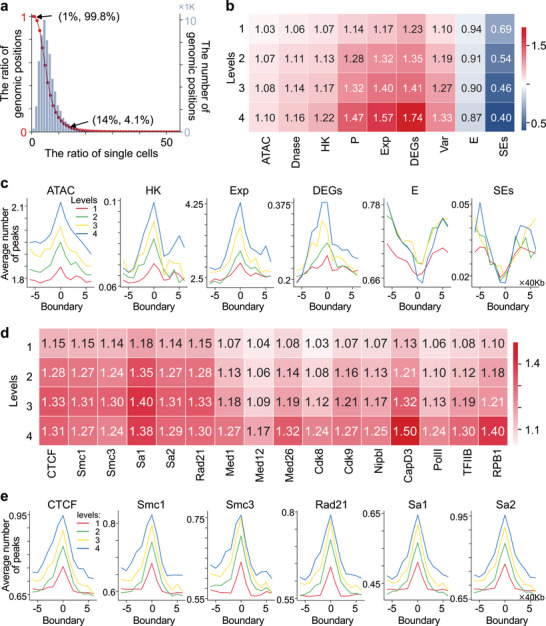
Regulatory factors navigate the preference of genomic position forming boundaries. a) The ratio distribution of genomic positions forming boundaries at less than a given ratio of single cells in the left *y*‐axis (such as 99.8% of genomic positions form boundaries in at least 1% of cells, respectively), and the number distribution of genomic positions forming boundaries in the right y‐axis as the ratio of single cells increases. b,c) The concentration scores (b) and the average number of ATAC‐seq peaks and multiple regulatory elements (ATAC: ATAC‐seq peaks, HK genes, Exp: gene expression value, DEGs: differentially expressed genes, E: enhancers, SEs) (c) around domain boundaries. d) The concentration scores for CTCF, cohesin, mediator‐ and Pol II‐associated factors. e) The average number of peaks for CTCF and cohesin around domain boundaries. In (b)–(e), the results were detected in the different genomic scales across all single cells. The concentration score is defined in Supporting Information.

The median size of chromatin domains increases and the median boundaries’ insulation strength enhances with the increase of genomic position level (Figure S2a–c, Supporting Information). The boundaries show obvious cell‐to‐cell heterogeneity with a nonzero probability of being located at any genomic position. 99.8% of genomic positions form boundaries in at least 1% of cells (**Figure** **3**a). We also observe that the probability of forming boundaries enhances with the levels of genomic position increasing (Figure S2d, Supporting Information). The above observations suggest that domain boundaries vary from cell to cell with nonzero probability at all genomic positions as reported in ref. [8a] and the preference of genomic position forming boundaries may shape the formation of hierarchical chromatin domains in single cells.

### 2.4 Regulatory Factors Navigate the Preference of Genomic Position Forming Boundaries

A recent study has shown that domain boundaries are preferentially located at CTCF‐ and cohesin‐binding sites with a super‐resolution chromatin tracing method.^[^ ^8a^ ^]^ CTCF and cohesin have been proven to be key factors controlling the functional architecture of mammalian chromosomes forming TADs or sub‐TADs by “loop extrusion”.^[^ ^12^ ^]^ Indeed, we observe that both CTCF and cohesin show similar enrichment patterns in the boundaries of single cells in different genomic levels, and as the level of boundaries increases, the degree of enrichment gradually increases (Figure 3d,e).

The previous study has illustrated that high levels of transcription activity may contribute to TAD formation in bulk analysis.^[^ ^13^ ^]^ Here, we observe that the accessibility of genomic positions, the enrichment degree of HK genes and differentially expressed genes (DEGs), and the expression of genes gradually increases along with the increasing levels of genomic positions (Figure 3b,c and Figure S2e, Supporting Information). Conversely, enhancers and super‐enhancers greatly become more absent with the increase of levels (Figure 3b,c). It suggests that the emergence of highly transcriptional activity, especially DEGs, and the absence of enhancers, especially SEs, improve the probability of genomic positions forming boundaries of single cells (Figure 3b,c).

Although the CTCF‐cohesin complex is critical for the formation of TADs in mammalian cells, a substantial number of boundaries remain unaffected after cohesin degradation in single cells, suggesting other modulators exist on domain boundaries.^[^ ^4^, ^14^ ^]^ We indeed observe that mediators (*Med1*, *Med12*, *Med26*, *Cdk8*, *Cdk9*), *Nipbl*, PolII, and TFIIB are all enriched in the boundaries of single cells, and as the level of boundaries increases, the degree of enrichment gradually increases (Figure S2f,g, Supporting Information). Mediators are essential coactivators that are recruited to the regulatory regions of active genes and facilitate the ability of enhancer‐bound TFs to recruit PolII to the promoters of target genes, and *Nipbl* has been proven to bind mediator to load cohesin.^[^ ^6^, ^15^ ^]^ The above results suggest that these factors may play important roles in shaping the preference of genomic position forming boundaries.

The master factors of Polycomb repressive complex 1 (PRC1) and PRC2 just exhibit two different enrichment patterns at the domain boundaries of single cells. One type (*Aebp2*, *Rybp*, and *Ring1b*) forms obvious single peaks at the domain boundaries, and the degree of enrichment gradually increases with the boundary level increasing (Figure S3a, Supporting Information). Another type (*Ezh2*, *Pcl2*, *Suz12*, and *Eed*) shows double peaks around the domain boundaries (Figure S3b, Supporting Information). These two types of complexes have been proven to have distinct catalytic activities, but both are generally associated with transcriptional silencing.^[^ ^16^ ^]^ We also observe that TrxG‐associated proteins (COMPASS: *Set1a*, *Mll2*, *Mll3/4*, and SWI/SNP: *Brg1*) are all enriched in domain boundaries, except for *Mll3/4* (Figure S3c, Supporting Information). In mammalian, *Set1a* reportedly contributes to most of the H3K4me3, and *Mll2* mediates H3K4me2 and H3K4me3 at developmental genes, while *Mll3/4* implements monomethylation of H3K4 at enhancers.^[^ ^17^ ^]^ *Brg1* is an ATP‐dependent chromatin remodeler, contributing to the maintenance of pluripotency and self‐renewal in ESCs.^[^ ^6a^ ^]^ The above results suggest that the PRC and TrxG protein families could change focal chromatin interactions in different ways.

We find that the core TFs (*Oct4*, *Sox2*, and *Nanog*) controlling the pluripotent state do not prefer to appear in domain boundaries, which may be related to chromatin hubs occupied by super‐enhancers/enhancers (Figure S4a, Supporting Information) as reported in ref. [18]. In addition, we also collect 14 additional TFs that may contribute to the pluripotent state of mESCs and investigate whether they are enriched in domain boundaries of single cells^[^ ^6a^ ^]^ (Figure S4b,c, Supporting Information). The results indicate that nine additional TFs (*Esrrb*, *Nr5a2*, *Klf4*, *Zfp281*, *Tcf3*, *Tcfcp2l1, Stat3*, *Prdm14*, and *Smad2/3*) that were previously shown to occupy both typical enhancers and SEs do not prefer to appear in domain boundaries, while five factors (*c‐Myc*, *n‐Myc*, *Zfx*, *Tbx3*, and *Yy*1) that were previously shown to occupy promoter‐proximal sites are enriched in domain boundaries.^[^ ^6a^ ^]^ Among them, it is particularly interesting that *Smad2/3*, *Stat3*, and *Tcf3* signaling pathways were considered key modulators controlling mESCs pluripotent state transition by modifying chromatin states and shaping chromatin domains.^[^ ^19^ ^]^

In addition, we also observe additional 45 proteins are either enriched or absent in domain boundaries of single cells with different enrichment patterns (Figures S5–S7, Supporting Information), which suggests their potential in shaping chromatin domains.

The above TFs and chromatin regulators have the most profound impact on cell states through collaborative control of chromatin states and spatial structures. 14 different histone‐modifying enzymes show a variety of enrichment patterns around domain boundaries of single cells (Figure S8, Supporting Information). For example, ZC3H11A shares consistent enrichment patterns with CTCF‐cohesin (Figure 3e and Figure S8, Supporting Information). H3K27me3 shares similar enrichment patterns with PRC2, while H2AK119ub1 shares similar enrichment patterns with PCR1 (Figure S3a,b, Supporting Information), which are consistent with their function in participating chromatin modifications.^[^ ^16^ ^]^ H3K4me1 marking of enhancers is not enriched in domain boundaries. H3K4me2 shares similar enrichment patterns with H3K4me3 around domain boundaries, both of which are associated with TrxG protein family ^[^ ^17^ ^]^ (Figure S3c, Supporting Information). It suggests different histone modifications may cooperate with different TFs and chromatin regulators, to modify chromatin states, shape the local chromatin interaction status and organize chromatin domains of single cells.

To sum up, we have observed hundreds of TFs, chromatin regulators, and histone modifications are significantly either enriched or absent in domain boundaries of single cells with differential enrichment patterns. The occupancy of these regulatory factors in specific genomic positions will affect focal chromatin interactions, thereby changing the interaction density or insulation strengths of these regions. These processes may navigate the preference of genomic position forming boundaries, and then shape hierarchical chromosome domains of single cells.

### 2.5 Cooperation among Regulatory Factors Differentiate Genomic Position Categories

**Figure 4:**
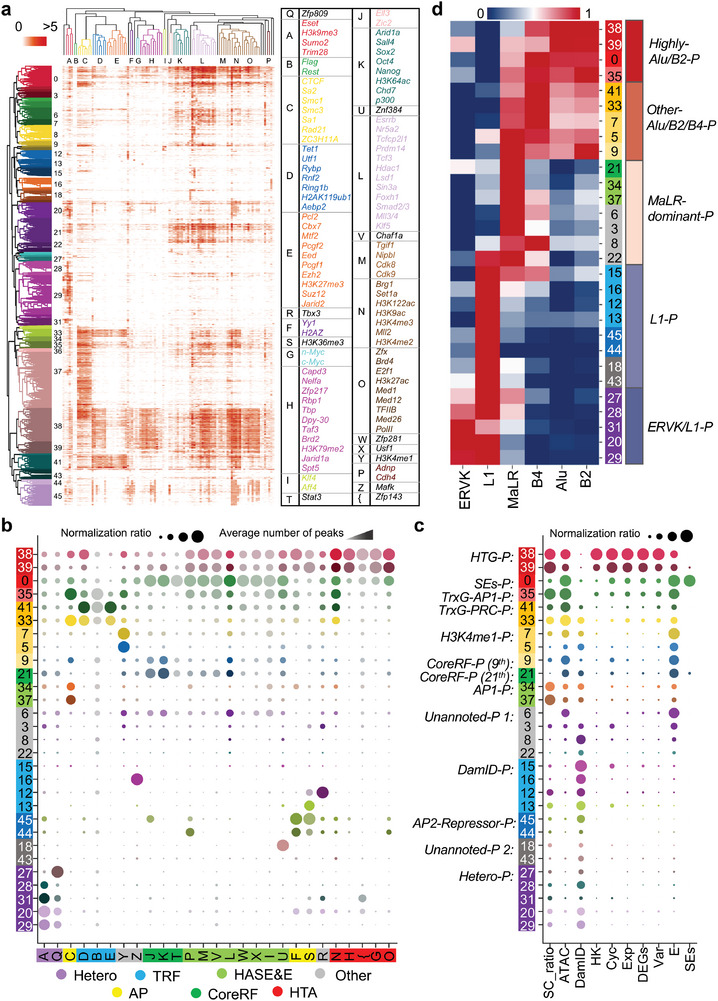
The systematic analysis on genomic position categories. a) The bi‐cluster of chromatin positions and regulatory factors. b) The column normalization ratio and the average number of peaks for regulatory factor classes on genomic position categories (different dot color represents different categories in (a). c) The column normalization ratio of different elements or factors, including SC_ratio (the ratio of single cells forming boundaries), ATAC, DamID (DamID‐seq), HK genes, Cyc (mark genes for cell cycle), Exp (gene expression value), DEGs, Variable (variable scores for genes), E (Enhancers), and SEs, on genomic position categories. d) The heatmap shows the column normalization ratio of different retrotransposons based on genomic position categories.

To further elaborate on cooperation patterns between different types of regulatory factors, and genomic position categories with differential preference forming boundaries driven by these cooperation patterns, we grouped 13 large spatially organized genomic position categories (consisting of 29 sub‐categories), which were annotated by seven different regulatory factor clusters (consisting of 27 sub‐clusters). The result helps to explain the preference of genomic position forming boundaries in single cells, and provides an increasingly complex view of the genomic structure‐function relationship (**Figure** **4** and Figure S9, Supporting Information).

#### 2.5.1 The Categories of Regulatory Factors

The result of hierarchical clustering indicates that these categories mainly cnsist of the cluster of core regulatory factors of mESC (CoreRF, particularly *Oct4*, *Sox2*, and *Nanog)*, the clusters associated with highly active activators for SEs and enhancers (HASE&E), the clusters of highly transcribed activators (HTA), the clusters of transcriptional repressor factors (TRF), the cluster associated with heterochromatin factors (Hetero), and the clusters of architectural proteins (AP). There are also a few single factors (Other) (Figure 4a and Figure S9, Supporting Information). For a specific example with fewer prior studies among these clusters, *Sumo2* is required to play critical roles in the canonical *Zfp809/Trim28/Eset* complex via post‐translational sumoylation of *Trim28*, which enhances the recruitment of *Trim28* to the proviral DNA, resulting in the modification of proviral chromatin with repressive histone H3K9me3 mark in turn.^[^ ^20^ ^]^ These four factors were grouped with H3K9me3 together in the analysis below, which may organize the formation of heterochromatin (Figure 4a). The annotation information and supporting materials of these categories were summarized in Table S2, Supporting Information.

We also observe some interesting cooperative patterns among different clusters (Figure S9b, Supporting Information). For example, the CoreRF cluster is strongly associated with the HASE&E cluster, but not the HTG cluster, while the HASE&E cluster is intimated to the HTG cluster. It suggests the HASE&E cluster may be a bridge between CoreRF and HTG clusters, which can be linked to various signaling pathways involved in the transcriptional network in mESCs.

#### 2.5.2 The Division of Genomic Position Categories

**Figure 5:**
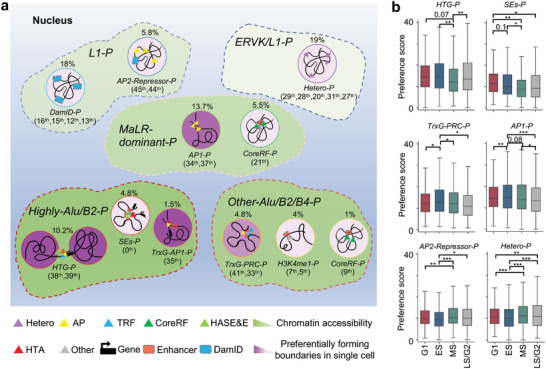
A schematic showing different genomic position categories. a) The graph presents the different clusters based on retrotransposons in the area marked by different colors of dotted lines containing multiple functional groups associated with different regulatory factor classes (the sub‐categories serial number above and the ratio occupying from all positions are marked for each functional group). The shades of purple on the background of circles indicate the preference of genomic positions forming boundaries in single cells for each functional group, and the shades of green on the background of these areas marked by a dotted line indicate the degree of chromatin accessibility of genomic positions for each cluster based on retrotransposons. b) Preference scores of different chromatin landscape categories across different cell states. Statistical significance is calculated by Welch's *t‐*test (*p < 0.05, **p < 0.01, and ***p < 0.001). The preference score is defined in Supporting Information.

We further analyze the preference of genomic position categories with a ratio >1% (Figure S10a, Supporting Information). These genomic position categories can be divided into three families, including the categories with high accessibility preferentially forming boundaries, the categories with high accessibility unfavorably forming boundaries, and the categories with low accessibility unfavorably forming boundaries (**Figure** **5**a and Figure S10b, Supporting Information).

Of note, the largest type with open chromatin accessibility (13.7% of all positions, consisting of 12.6% and 1.1% from the 37th and 34th classes, respectively, defined as AP1‐Positions) preferentially forming boundaries only are significantly occupied by AP1 factors (CTCF, cohesin, etc.) comparing with other types of factors (Figures 4b,c and 5a). Previous studies have indicated that loop extrusion executed by CTCF and cohesin is a leading factor governing domain formation and facilitating chromatin folding. We find that the other subunits (*Sa1* and *Sa2*) of cohesin occupy the same genomic locus and present similar enrichment patterns at the boundaries of single cells, suggesting that both *Sa1* and *Sa2* may also participate in the maintenance of domain boundaries (Figure 3e). ZC3H11A, a zinc finger protein, shows a uniform pattern as *Sa1* and *Sa2*, implying its novel role in shaping boundaries of chromatin domains (Figure S8, Supporting Information). The chromatin positions that AP1 proteins occupy may be directly related to the macroscopic architecture of chromatin within the nucleus, and indirectly change the local chromatin context to exert regulatory functions.

The second major category of open chromatin positions preferentially forming boundaries (7.5% and 2.7% from the 38th and 39th classes, defined as HTG‐Positions) is associated with highly transcribed genes (Figures 4b,c and 5a). These regions are mainly occupied by the HTA (particularly the TrxG class, playing important roles in orchestrating the stable activation of gene expression) and HASE&E clusters, but not the CoreRF cluster, which indicates that the CoreRF cluster hardly participates in the regulation of genes occupying in domain boundaries of single cells.

The third major category of open chromatin positions preferentially forming boundaries (3.1% and 1.7% from the 41^th^ and 33^rd^ classes, defined as TrxG‐PRC‐Positions, 1.5% from the 35^th^ class, defined as TrxG‐AP1‐Positions) is associated with PRC, TrxG, and AP1 proteins (Figures 4b,c and 5a). The 33^rd^ class of genomic regions is also associated with AP1 proteins with a higher probability of forming domain boundaries of single cells compared with the 41^st^ class, which suggests CTCF‐cohesin complexes may help these regions form a more stable structure. In addition, the 35^th^ class of genomic region with higher enrichment of TrxG proteins and lower enrichment of PRC proteins compared with the 33^rd^ class, which results in higher open chromatin accessibility, enhancers occupation, and probability forming domain boundaries in single cells. The above results indicate that chromatin modifiers (TrxG and PRC proteins) can provide an additional layer of regulation by changing chromatin structures, and balance the formation of domain boundaries by repressing and activating chromatin states, respectively.

The major categories of the open chromatin positions unfavorably forming boundaries are associated with highly active SEs or enhancers, which mainly contain three categories (Figures 4b,c and 5a). The first category (4.8% from the 0^th^ class, defined as SEs‐Positions) almost encompasses more than 75% of SEs, which are strongly related to the HASE&E, CoreRF, TrxG, and Med‐PolII clusters. The second category (5.5% and 1% from the 21^st^ and 9^th^ classes, defined as CoreRF‐Positions) is mainly associated with enhancers occupied by CoreRF. The slight difference between the 21^st^ and 9^th^ classes is that the 9^th^ one shows higher chromatin accessibility, while the 21^st^ one shows a stronger DamID enrichment, which may be related to their locations in the nucleus. The third category (2.7% and 1.3% from the 7^th^ and 5^th^ classes, H3K4me1‐Positions) is related to enhancers enriched by H3K4me1. These regions taking SEs or enhancers associated with CoreRF and H3K4me1 as focal regions generate dense chromatin structures mediated by different regulatory factors in an orientation‐independent manner, and unfavorably form boundaries in single cells with weakened insulation strengths.^[^ ^21^ ^]^

We also check the classes of genomic positions with low chromatin accessibility, all of which unfavorably form boundaries in single cells. These chromatin positions are mainly divided into the following categories (Figures 4b,c and 5a). The first category (19% of all positions, consisting of 9.2%, 3.7%, 3.5%, 1.6%, and 1% from the 29^th^, 28^th^, 20^th^, 31^st^, and 27^th^ classes, respectively, defined as Hetero‐Positions) are associated with the Hetero cluster. The second category (18% of all positions, consisting of 2.8%, 2.3%, 1.9%, and 1.7% from the 16^th^, 15^th^, 12^th^, and 13^th^ classes, respectively, defined as DamID‐Positions) maintains high enrichment of the DamID signal. The above observations suggest that the presence of heterochromatin factors reduce the probability of single‐cell domain boundary formation. Finally, we observe an interesting and specific category (4.6% and 1.2% from the 45^th^ and 44^th^ classes, defined as AP2‐Repressor‐Positions), which are associated with the AP2 (*Yy1* and H2AZ) and H3K36me3 classes (Figures 4a and 5a). Both *Yy1* and H2AZ facilitate the organization of genome architecture (Table S2, Supporting Information).^[^ ^22^ ^]^

To summarize, we obtain the following key results by the above analysis: 1) The genomic positions occupied by architectural proteins (CTCF and cohesin), highly transcribed genes, and TrxG proteins preferentially form boundaries in the single cells analysis. 2) The genomic positions taking SEs or enhancers associated with CoreRF or marked by H3K4me1, heterochromatin factors, and repressing factors unfavorably form boundaries in single cells with weakened insulation strengths (Figure 5a and Table S3, Supporting Information).

### 2.6 Retrotransposons Are Associated with These Genomic Position Categories

In a recent paper, Shen and colleagues find that retrotransposons embedded in 3D genome architecture, regulate the formation of euchromatin and heterochromatin respectively, particularly the separation of compartments A/B,^[^ ^23^ ^]^ which are consistent with our observations. Notably, our study focuses on the effect of retrotransposons on the preference of genomic position forming boundaries in single cells. In order to explain the preference of genomic position categories forming boundaries in single cells with more detail, we further analyze the influences of the largest six types of retrotransposons on genomic architecture.

We observe that the division of genomic position categories is strongly associated with the regulation of retrotransposons. The genomic positions are clearly divided into five functional units based on categories of retrotransposons, including Highly‐Alu/B2‐Positions, Other‐Alu/B2/B4‐Positions, MaLR‐dominant‐Positions, L1‐Positions, and ERVK/L1‐Positions (Figures 4d and 5a).

Firstly, all genomic regions (Hetero‐Positions, DamID‐Positions, and AP2‐Repressor‐Positions) with low accessibility and unfavorably forming boundaries are enriched by L1 elements, which tend to occupy gene‐poor, heterochromatic B compartments that interact with lamina‐associated domains in previous studies.^[^ ^23a^ ^]^ Among the defined regions, we are surprised to find that Hetero‐Positions are specially associated with ERVK elements, which may indicate that ERVK acts as specific roles in regulating embryonic development as reported in.^[^ ^24^ ^]^ The above observation also implies that the previously unannotated 18^th^ and 43^rd^ genomic positions may be related to heterochromatin organization.

We also observe that genomic regions (HTG‐Positions, SEs‐Positions, TrxG‐AP1‐Positions, TrxG‐PRC‐Positions, H3K4me1‐Positions, the 9^th^ class of CoreRF‐Positions) are enriched by Alu/B2/B4, which may be related to euchromatin organization.^[^ ^23a^ ^]^ HTG‐Positions, SEs‐Positions, as well as TrxG‐AP1‐Positions with higher Alu/B2 enrichment than others, suggest that the enrichment of Alu/B2 may indicate the transcription level of genes, and promote the formation of hierarchical chromatin structures by regulating gene transcription and SEs/Enhancer activation.

Besides the above genomic positions, what is interesting is that MaLR elements are enriched in AP1‐Positions, the 21^st^ class of CoreRF‐Positions, and other unannotated regions (the 6^th^, 3^rd^, 8^th^, and 22^nd^ classes). We first observe that the 21^st^ class occurs a stronger heterochromatin factors (the Hetero cluster) enrichment compared to the 9^th^ (CoreRF‐Positions), which may result in uncertain chromatin states in the 21^st^ position (Figure 4b,c). In addition, previous studies have shown that domain boundaries mediated by AP1 proteins (e.g., CTCF and cohesin) may block the spread of chromatin states.^[^ ^2^ ^]^ It may suggest that the regions dominantly enriched by MaLR elements may often undergo switches between euchromatin and heterochromatin.

In summary, we find that: 1) L1‐Positions with low accessibility and unfavorably forming boundaries are associated with heterochromatin organization, and Alu/B2/B4‐Positions are associated with euchromatin chromatin, which are consistent with a recent study,^[^ ^23a^ ^]^ while MaLR‐Positions may result in switches between euchromatin and heterochromatin, which is yet to be proven. 2) ERVK elements act as specific roles in heterochromatin formation, while Alu/B2 may promote high transcription of genes and high activation of SEs/Enhancers (Figure 5a and Table S3, Supporting Information). These retrotransposons contribute to the maintaining of chromatin states, and interplay with other types of regulatory factors, to navigate the preference of genomic positions forming boundaries and gene regulation in single cells.

### 2.7 Genomic Landscape Regulates Cellular States

To investigate the preference of genomic positions in the above functional groups along the cell cycle process, we check the dynamics of each functional group forming boundaries in single cells among four different cycle phases, including G1, early‐S (ES), mid‐S (MS), and late‐S/G2 (LS/G2). We first reveal that genomic positions enriched by SEs show a significant preference for forming boundaries in the G1 phase (Figure 5b). The observation suggests that high activation of SEs in the phase may promote gene regulation and transcription for cell growth in size, and ensure biomaterials for DNA synthesis. In addition, the functional group (HTG‐Positions) accompanied by highly transcribed genes exhibits a significant loss of boundaries in the MS phase, which may be because the rates of transcription and protein synthesis are low during DNA replication (Figure 5b). We also observe that functional groups occupied by both CTCF‐cohesin and TrxG‐PRC complexes prefer to form boundaries in ES phases (Figure 5b). The observation implicates the clearest segmentation of chromatin structures at the beginning of DNA replication.^[^ ^25^ ^]^ Both complexes have been proven to modify local chromatin structure and regulate higher‐order chromatin organization.^[^ ^4^, ^26^ ^]^ And functional groups (Hetero‐Positions and AP2‐Repressor‐Positions) associated with heterochromatin organization prefer to form boundaries in both MS and LS/G2 phases (Figure 5b). Both phases may prepare for everything entering the mitosis phase with condensing chromatin states.

In general, genomic positions (HTG‐Positions, SEs‐Positions, TrxG‐AP1‐Positions, TrxG‐PRC‐Positions, H3K4me1‐Positions, and the 9^th^ class of CoreRF‐Positions) enriched by Alu/B2/B4 retrotransposons have higher preference scores for forming boundaries of single cells in G1 and ES phases in comparison with MS and LS/G2 phases, whereas genomic positions (Hetero‐Positions, AP2‐Repressor‐Positions, and DamID‐Positions) enriched by L1/ERVK retrotransposons display opposite tendency, following by high preference scores for forming boundaries in both MS and LS/G2 phases (Figure 5b and Figure S11, Supporting Information). The above observations further expound that the dynamic interplay among different types of regulatory factors, retrotransposons, and chromatin structures could navigate gene regulation and cell functions, even cell identity in single embryonic stem cells.

## 3 Discussion

Several decades of research have shown that eukaryotic chromatin adopts a complex hierarchical architecture within the nucleus, which plays a key role in functional implications for almost all nuclear processes. Thus, the spatially organized chromatin architecture interplaying with multiple types of regulatory factors shapes focal chromatin landscapes and then exerts gene regulatory functions. Single‐cell 3D genome analysis extends the limitation of bulk analysis to show substantial cell‐to‐cell variation and promote our understanding of chromatin structures in the individual cell. Recent discoveries on single‐cell 3D genomes have shed light on the relationship between CTCF‐cohesin complexes and domain formation, but the molecular details associated with regulatory factors remain to be investigated.^[^ ^8a^ ^]^

Here, we develop HiCS to detect hierarchical chromatin domains from single‐cell Hi‐C maps and observe hundreds of regulatory factors, including TFs, chromatin regulators, and histone modifications, are significantly either enriched or absent in domain boundaries of single cells, which present several different enrichment patterns. The results suggest their potential cooperative associations in shaping focal chromatin interactions, thereby changing the interaction density or insulation strength of these regions, and driving different genomic position categories. We further group chromatin position categories and different regulatory factor clusters, explaining the emergence and functionality of different chromatin landscapes and providing a comprehensive view of the genomic structure‐function relationship. We also find that different retrotransposons exactly match the above genomic position categories. In summary, differential distribution of functional elements in specific genomic positions interplay with different cooperation patterns of regulatory factors will affect focal chromatin interaction, thereby changing interaction density and insulation strength of these regions, which may navigate the preference of genomic positions forming boundaries, shape hierarchical chromatin architecture, and then regulate cell functions, even cell identity in single embryonic stem cells (Table S3 and Figure S12, Supporting Information).

The chromatin structures within the nucleus operate in an obvious dynamic process driven by both “loop extrusion” and an attractive process induced by regulatory factors (associated with compartmentalization). The process may condense or lose local chromatin landscapes in an overlapping and concerted manner accompanied by adjusting the insulation strength of chromatin position and generating chromatin loops, and then shaping gene expression programs during cell‐fate specification.^[^ ^25a^ ^]^ Further work is needed to leverage more specific chromatin structures, particularly chromatin loops, of single cells with more abundant regulatory factors (TFs, chromatin regulators, histone modifications, retrotransposons, RNA, and even structural variations) to understand structure‐function relationships in complex tissues or diseases, particularly cancers.^[^ ^27^ ^]^ It will promote our understanding of how multiple types of regulatory factors interact with chromatin topological engines (such as loop extraction and compartmentalization) to regulate the gene‐repression program, determine cell functions and identity, and further explain tissue complexity and disease development.

## 4 Experimental Section

### Single‐Cell Hi‐C Data Generated from mESCs

The single‐cell Hi‐C dataset used in this study consisted of 1992 diploid cells of mESCs grown in 2i media without feeder cells with a stringent quality control filter. This dataset involved a median number of 393 506 restriction fragments, and 127 233 distinct > 1 kb contacting pairs on average per cell.^[^ ^11^ ^]^ The top 1315 cells with > 250 000 contacts per cell were selected for downstream analysis. Among them, 317, 341, 303, and 354 cells belonged to G1, early‐S (ES), mid‐S (MS), and late‐S/G2 (LS/G2) phases labeled by fluorescence‐activated cell sorting (FACS) sort criterion, respectively.

### An Atlas of ChIP‐seq for mESCs

An atlas of ChIP‐seq for hundreds of regulatory factors of mESCs was organized (Table S1, Supporting Information), including CTCF, cohesin (*Smc1, Smc3, Rad21, Sa1, Sa2*), mediators (*Med1, Med2, Med26, Cdk8, Cdk9*), codensin (*Capd3, Nipbl*), PolII, TFIIB, polycomb repressive complex (*Aebp2, Rybp, Ring1b, Rnf2, Suz12, Ezh2, Eed, Pcl2*), trithorax protein family (*Set1a, Mll2, Mll3/4, Brg1*), the core regulatory factors of mESC (*Oct4, Sox2, Nanog*), the regulatory factors of ESC occupied on enhancers or SEs (*Esrrb, Nr5a2, Klf4, Stat3, Prdm14, Zfp281, Tcf3, Tcfcp2l1, Smad2/3*), the regulatory factors of ESC occupied on promoter‐proximal sites or sites that border topological domains (*c‐Myc, n‐Myc, Zfx, Tbx3, Yy1*), and additional 45 TFs. 14 histone modification factors, including H3K4me1, H3K4me2, H3K4me3, H3K27me3, H3K36me3, H3K79me2, H3k9me3, H3K9ac, H3K122ac, H3K64ac, H3K27ac, H2AZ, ZC3H11A, and H2AK119ub1 were collected. In addition, two chromatin accessibility datasets (ATAC‐seq and Dnase‐seq) and the DamID‐seq dataset were also collected.

For the ChIP‐seq of regulatory factors, peaks were called using MACS2 software with a *q‐*value cut‐off, 1 × 10^−5^.^[^ ^28^ ^]^ The source information and supporting materials of these factors were summarized in Tables S1 and S2, Supporting Information.

### Regulatory Elements and Genes

Enhancers/SEs and gene expression datasets of mESCs were downloaded in GSE29278.^[^ ^29^ ^]^ Housekeeping genes were downloaded in Housekeeping and Reference Transcript Atlas (HRT Atlas v1.0, www.housekeeping.unicamp.br).^[^ ^30^ ^]^ PhastCons scores were downloaded from the UCSC Genome Browser via www.hgdownload.cse.ucsc.edu/goldenPath/mm9/phastCons30way/vertebrate.^[^ ^31^ ^]^ Mouse cell‐cycle annotated genes were obtained from the mouse genome informatics (MGI) (http://www.informatics.jax.org/), containing 891 genes relating to cell cycle process and regulation. The authors adopted Seurat to detect DEGs and the variable score of genes using *FindAllMarkers* and *FindVariableFeatures* functions based on single‐cell RNA‐seq data of ESCs, which consists of 182 cells labeled by FACS sort criterion, including 59, 58, and 65 cells belonging to “G1”, “S”, and “G2M” phases, respectively.^[^ ^32^ ^]^

### Retrotransposons

Retrotransposons built from RepeatMasker annotations were downloaded from the UCSC Table Browser (http://genome.ucsc.edu/). The authors kept the top six categories of counts for the downstream analysis, including Alu, B2, B4, MaLR, L1, and ERVK.

### Preprocess Contact Probability for each Chromosome of the individual cell

Each chromosome was first divided into bins of specific size (40 kb in this study) and the contact was contacted for each bin pair. Next, the authors modeled each chromosome as an unweighted network (each bin is one node, and each bin pair with non‐zero contacts is added as one edge), and implemented a classic graph embedding method node2vec, which applied a biased random walks procedure, to compute the contact probability of edges by computing the cosine similarity of any two node embedding vectors, and obtained the preprocessed matrix *A* (Figure 1a).^[^ ^33^ ^]^ The authors only kept the top 5% pairs for downstream analysis and the diagonal pairs were removed in this study.

### Detect Domain Boundaries for each Individual Cell

Inspired by a fast density‐based clustering method designed for grouping data points,^[^ ^34^ ^]^ the authors took advantage of finding the cluster centers to detect domain boundaries for each chromosome of individual cells (Figure 1b,c). Specifically, two indexes were defined for each 40 kb bin: 1) insulation strength *ρ*(*i*) of the *i* ^th^ genomic position is defined as the ratio of (*I*_*i*,intra_ − *I*_*i*,inter_) and (*I*_*i*,intra_ + *I*_*i*,inter_)^[^ ^35^ ^]^ using an 800 kb sliding window size:

(1) $$I_{i,\mathrm{intra}}=I_{a}+I_{b}$$

(2) $$I_{i,\mathrm{inter}}=I_{c}$$

(3) $$\rho \left(i\right)=\frac{I_{i,\mathrm{intra}}-I_{i,\mathrm{inter}}}{I_{i,\mathrm{intra}}+I_{i,\mathrm{inter}}}$$

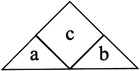


where *I_a_*, *I_b_*, and *I_c_*, respectively, represent the summation of interaction frequencies for regions *a*, *b*, and c, and 2) minimum distance between the bin *i* and any other bin *j* with higher insulation strength is defined as *δ*(*i*):

(4) $$\delta \;\left(i\right)=\;\min_{j:\rho \left(j\right)>\rho \left(i\right)}\left|i-j\right|,\;\left(\delta_{\max}<\mathrm{MAX}\right)$$

The authors searched for higher insulation strength of bin *i* in the range of *MAX* = 500 (20 M genomic distance at 40 kb resolution). Next, the authors defined *ρ*′(*i*) = *ρ*′(*i*)/*ρ*_max_ and *δ*′(*i*) = *δ*(*i*)/*δ*_*max*_ such that both *ρ*′(*i*) and *δ*′(*i*) were within range [0, 1]. Then, the rank *γ* of all bins was generated for each chromosome by their *η*(*i*) = *ρ*′(*i*) × *δ*′(*i*) in the descending and the rank of each bin was normalized by *γ*′(*i*) = *γ*(*i*)/*γ*_max_. The optimal reflection point was defined with $r=\underset{i=1:len}{argmin}\eta {\left(i\right)}^{2}+\gamma^{\prime }{\left(i\right)}^{2}$, where *len* is defined as the number of bins for a specific chromosome. The boundaries of the optimal structure were assigned by bins with *η*(*i*) > *α* × *η*(*r*), (*α* = 1). The gap regions were defined by *I*_*i*,*inter*_ = 0 or had no contact with any other bins.

### Determine the Hierarchical Chromatin Domains

The above procedures of detecting domain boundaries were run multiple times by defining *η*(*i*) > *α* × *η*(*r*) as a screening selection for different genomic levels, where the range of *α* was set as (0.1, 10) (Figure 2a). In this study, the authors selected multiple scaling parameters *α* as {0.2, 1, 4, 8} to obtain the hierarchical domains of chromatin, according to the distribution of the domain sizes and insulation strengths under different scaling parameters (Figure 2a,b).

### Clustering and Annotating Genomic Positions with Different Types of Regulatory Factors

The hierarchical clustering method was applied to group regulatory factors and chromatin positions, respectively (Figure 4a). The classes of chromatin positions with a ratio > 1% of all chromatin positions were selected for downstream analysis, which led to 29 classes of chromatin positions, along with 27 different transcriptional factor classes.

The hierarchical clustering method was further applied to merge the 27 classes into seven large clusters, based on the Pearson correlation of the normalization ratio of the mean counts for peaks of regulatory factor classes (Figure S9b, Supporting Information). These clusters or sub‐clusters were manually annotated based on the annotation information (Table S2, Supporting Information). Different chromatin position classes were then merged into 12 large categories based on hierarchical clustering of correlation of regulatory factor classes, and these categories were manually annotated based on the ratios of regulatory factor classes. The 12 large chromatin position categories (consisting of 29 sub‐categories) and seven different regulatory factor clusters (consisting of 27 sub‐clusters) were obtained and annotated.

### Data and Software Availability

All datasets analyzed in this study were published previously. The corresponding descriptions and preprocessing steps can be found in Supporting Information. The open‐source HiCS python package and tutorial are available at GitHub (https://github.com/YusenYe/HiCS).

## Conflict of Interest

The authors declare no conflict of interest.

## Author Contributions

Y.Y. conceived the idea, implemented the algorithm, and performed the analyses. Y.Y. interpreted the results. S.Z. and L.G. provided scientific insights on the applications. Y.Y. wrote the manuscript with feedback from all other authors. All of the authors read and approved the final manuscript.

## Supporting information

Supporting Information

## Data Availability

The data that support the findings of this study are available in the supplementary material of this article.
