# Supplementary material for: Deciphering Hierarchical Chromatin Domains and Preference of Genomic Position Forming Boundaries in Single Mouse Embryonic Stem Cells
Source: Adv Sci (Weinh). 2023 Jan 19;10(8):2205162. doi: 10.1002/advs.202205162 (PMC10015865; doi:10.1002/advs.202205162)
Supplement: Supplementary file 1 — Supporting Information [file ADVS-10-2205162-s001.pdf]

ADVANCED SCIENCE
Open Access

Supporting Information

for *Adv. Sci.*, DOI 10.1002/advs.202205162

Deciphering Hierarchical Chromatin Domains and Preference of Genomic Position Forming Boundaries in Single Mouse Embryonic Stem Cells

Yusen Ye, Shihua Zhang, Lin Gao, Yuqing Zhu and Jin Zhang*

Supporting Information

Deciphering Hierarchical Chromatin Domains and Preference of Genomic Position Forming Boundaries in Single Mouse Embryonic Stem Cells

Yusen Ye, Shihua Zhang, Lin Gao, Yuqing Zhu, Jin Zhang*

Supplementary Methods

The columns normalization ratio of different regulatory factors

The columns normalization ratio of the kth factor on the lth chromatin landscape class is defined as

$$CNR(k,l) = \frac{MC(k,l) - \min_{i=1,\dots,m} MC(k,i)}{\max_{i=1,\dots,m} MC(k,i) - \min_{i=1,\dots,m} MC(k,i)}$$

where $MC(k,i)$ represents the average number for peaks of the kth factor on the ith chromatin landscape class.

The concentration scores

The concentration scores of the ith regulatory factor class on domain boundaries across all single cells are defined as

$$CS(i) = \begin{cases} m(b=0) \big/ \min_{k=-6,\dots,6} m(b=k), & if\ m(b=0) \geq m(-l \leq b \leq l) \\ m(b=0) \big/ \max_{k=-l,\dots,l} m(b=k), & else \end{cases},$$

where $m(b=0)$ represents the average number of peaks on domain boundaries across all single cells and $m(-l \leq b \leq l)$ represents the average number of peaks on all ± 6 bins around domain boundaries across all single cells. $m(b=l)$ represents the average number of peaks on the $|k|^{th}$ bin of upstream or downstream of domain boundaries across all single cells.

The preference score of genomic positions across different cell states

The preference score of genomic position k is defined as $PS(k) = c_s^k * 10^6 / \sum_{i=1}^{n} c_s^i$, where c_s^i represents the counts of genomic position i for forming boundaries given one cell state s, and n is the number of genomic positions.

Supplementary Figures:

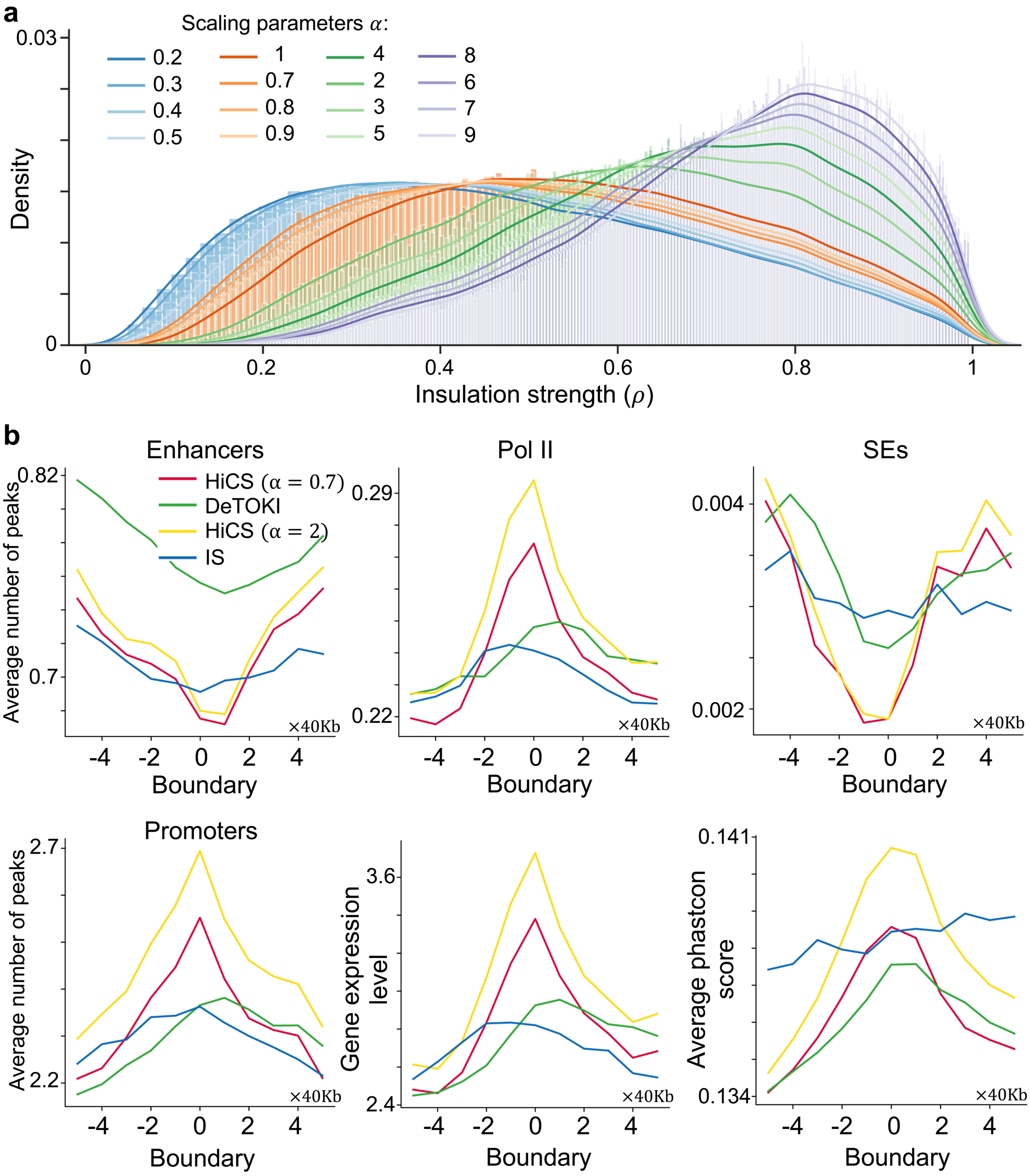

Supplementary Figure S1. a. The density distribution of insulation strengths at multiple scaling parameters. **b.** The average number of PolII peaks, enhancers, SEs, promoters, the gene expression level, and the mean phastcon score at domain boundaries of single cells. The above results were implemented by different methods or scaling parameters across all single cells.

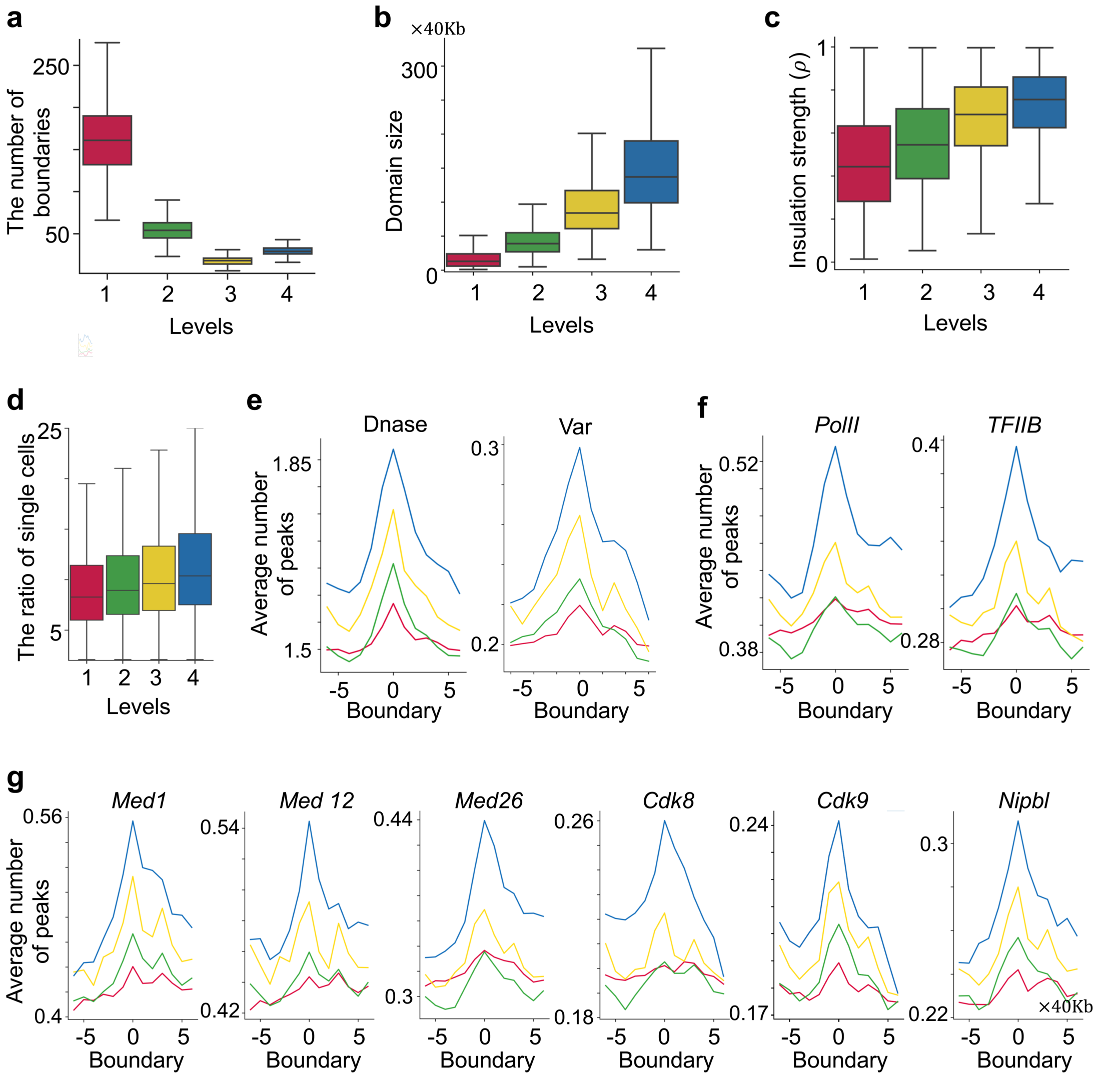

Supplementary Figure S2. a. The average number of boundaries. **b.** Domain size. **c.** Insulation strengths of boundaries. **d.** The ratio of single cells forming boundaries. In **a-d**, the results were detected in the different genomic scales across all single cells on Chromosome 2. **e-g.** The average number of Dnase-seq peaks, Var (variable scores for genes), and multiple regulatory factors (PolII- and mediator-associated factors) peaks across domain boundaries of all single cells in the different genomic scales.

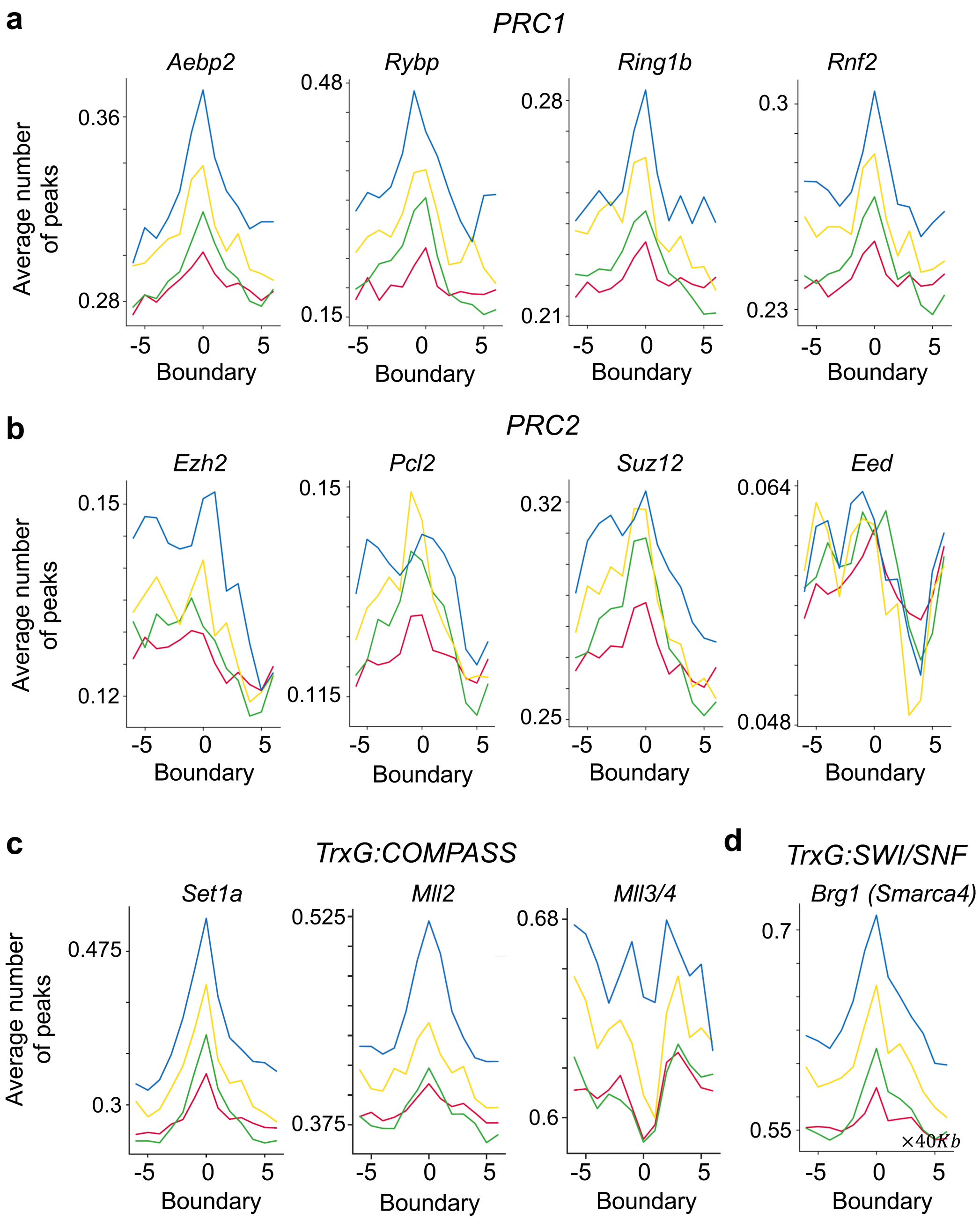

Supplementary Figure S3. a, b, c. The average number of peaks for the master Polycomb repressive complex 1 and 2(PRC1 (a) and PRC2 (b), respectively) and TrxG proteins (COMPASS and SWI/SNF) (c) across domain boundaries of all single cells in the different genomic scales.

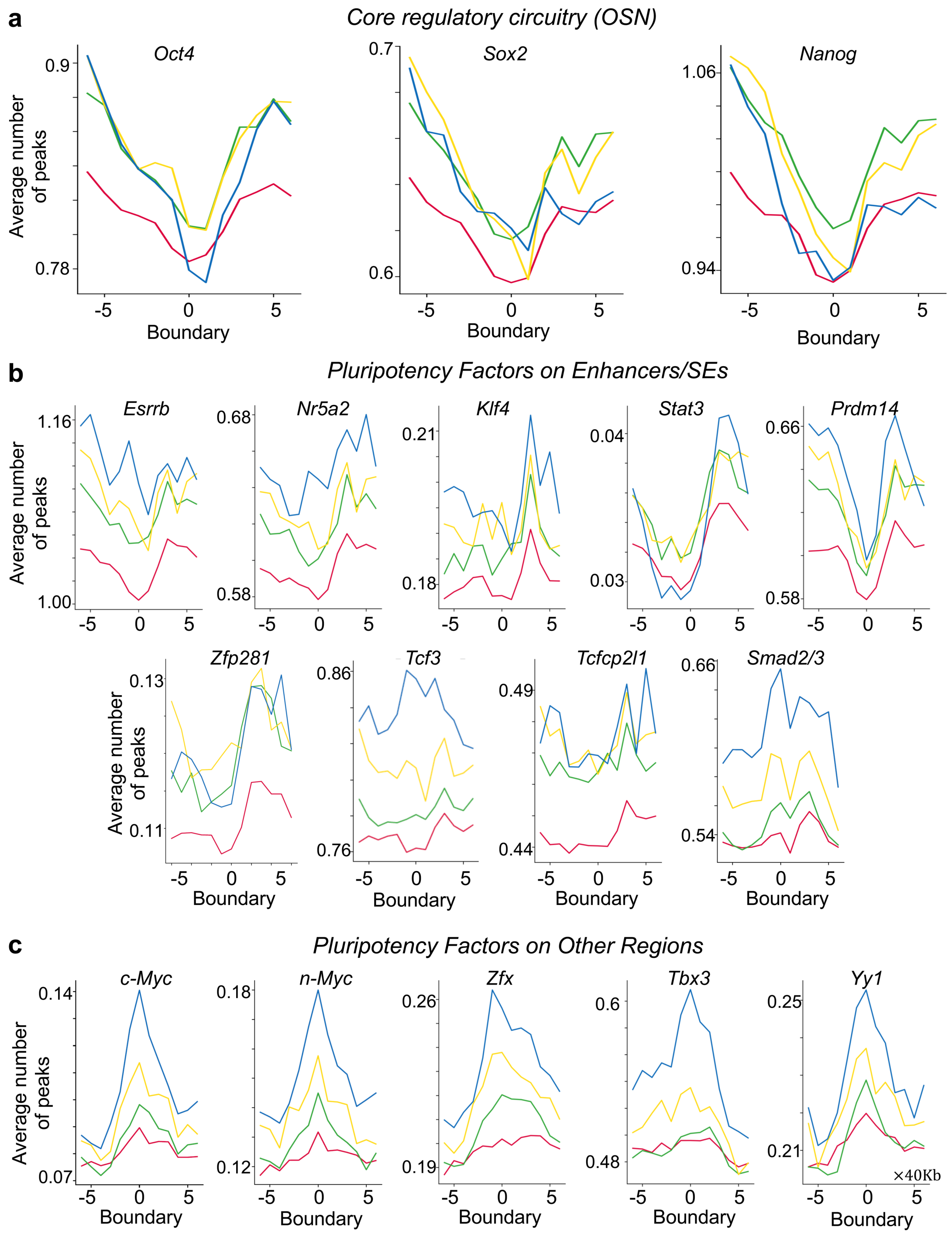

Supplementary Figure S4. a, b, c. The average number of peaks for the core regulatory circuitry factors (**a**), pluripotency factors enriched in enhancers/SEs (**b**), and pluripotency factors enriched in other regions such as promoter-proximal sites (**c**) across domain boundaries of all single cells in the different genomic scales.

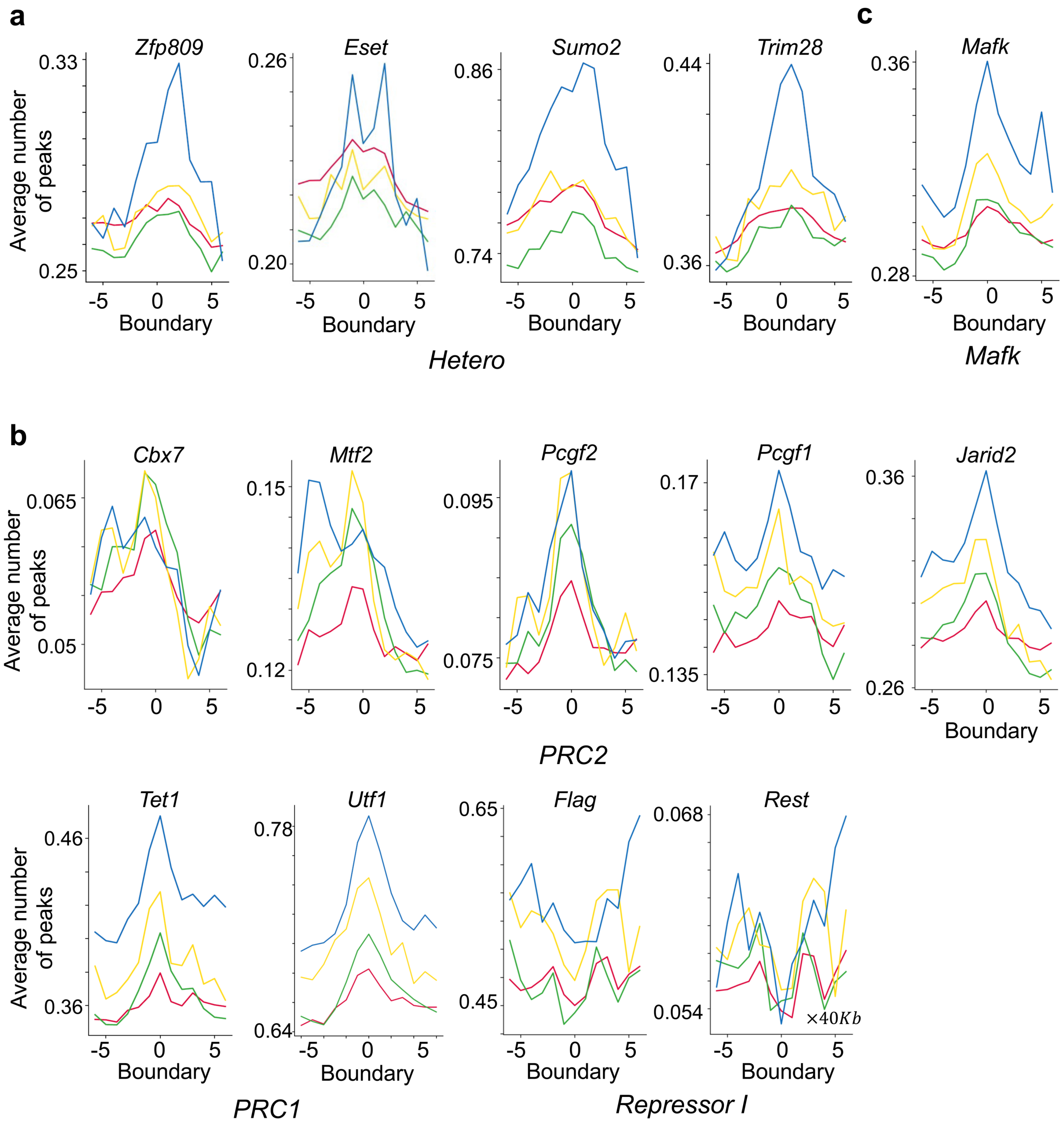

Supplementary Figure S5. The average number of peaks for multiple histone modifications across domain boundaries of all single cells at the different genomic scales. Each histone modifications correspond to different regulatory factors classes annotated in Fig. 4b.

Supplementary Figure S6

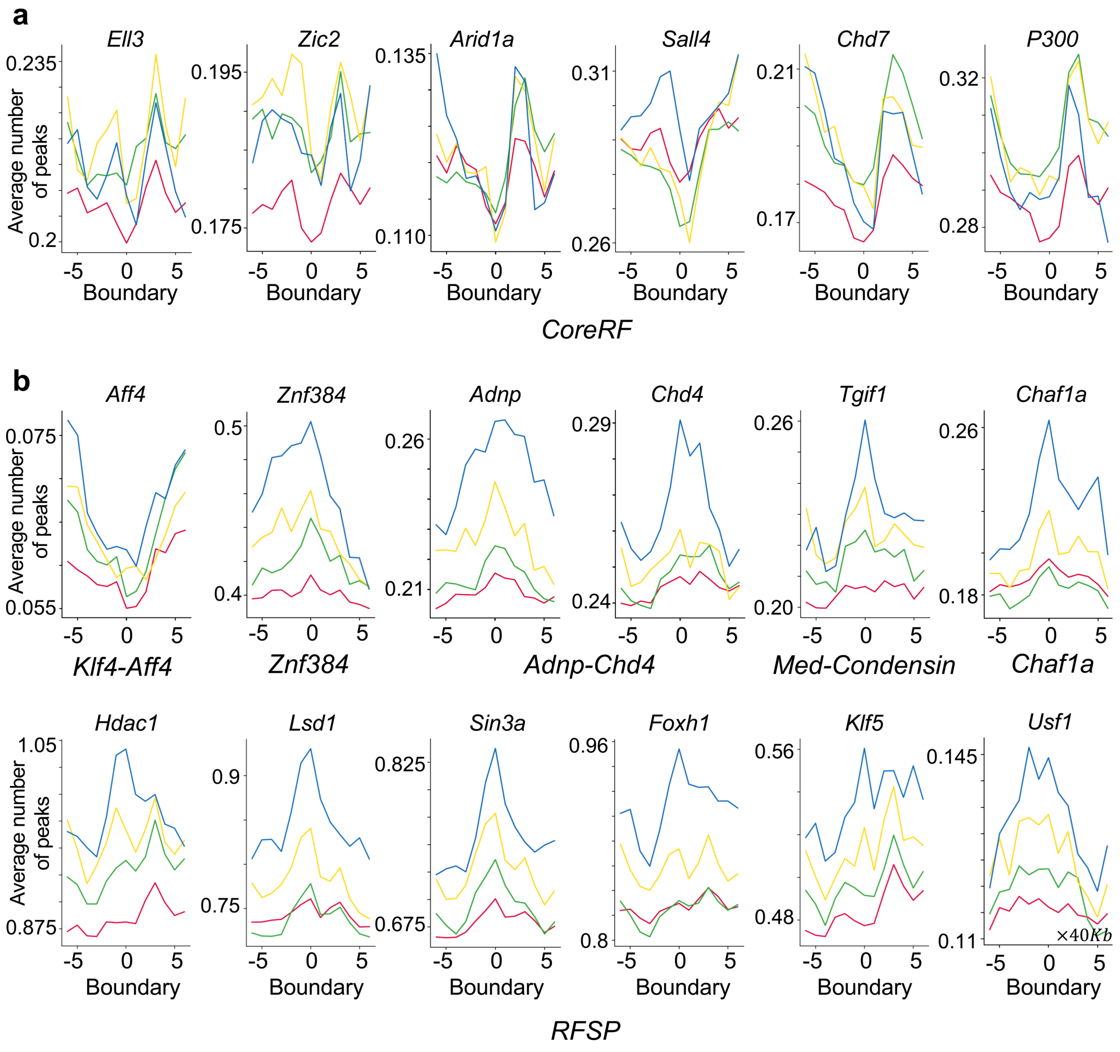

Supplementary Figure S7

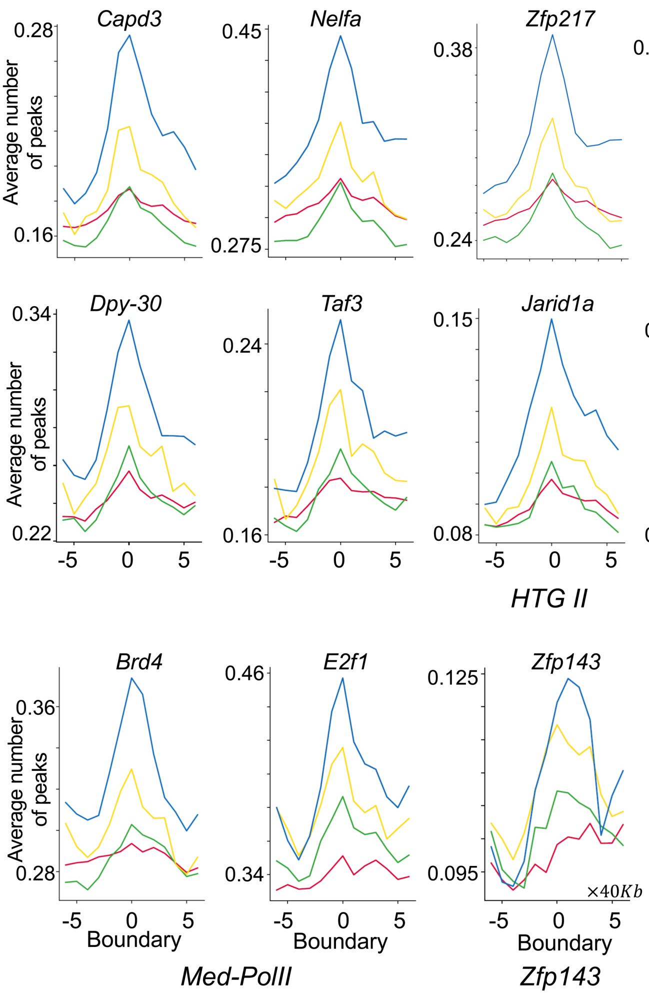

Supplementary Figure S8

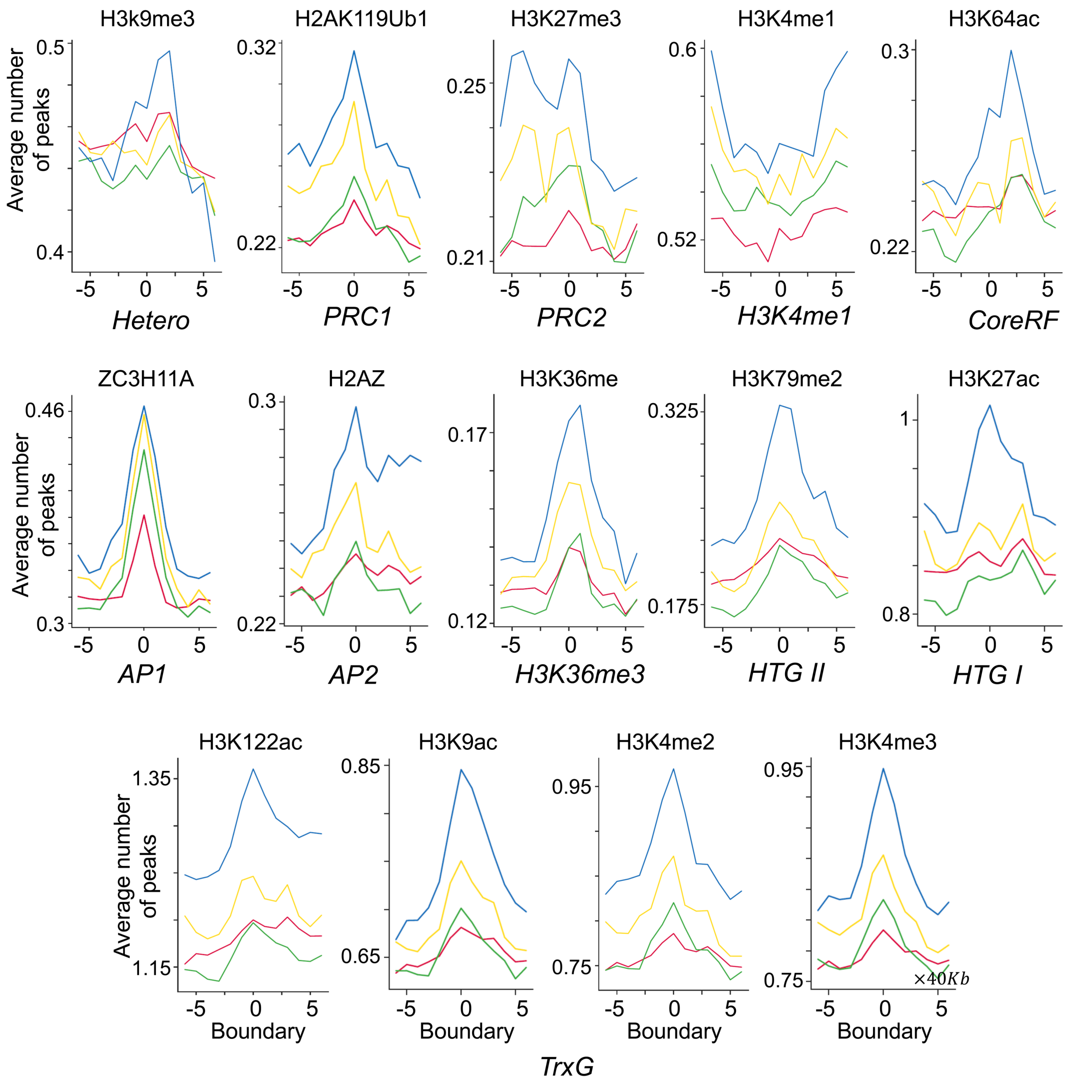

Supplementary Figure S6-S8. The average number of peaks for other regulatory factors across domain boundaries of all single cells in the different genomic scales. These factors include different classes of regulatory factors annotated in Fig. 4b.

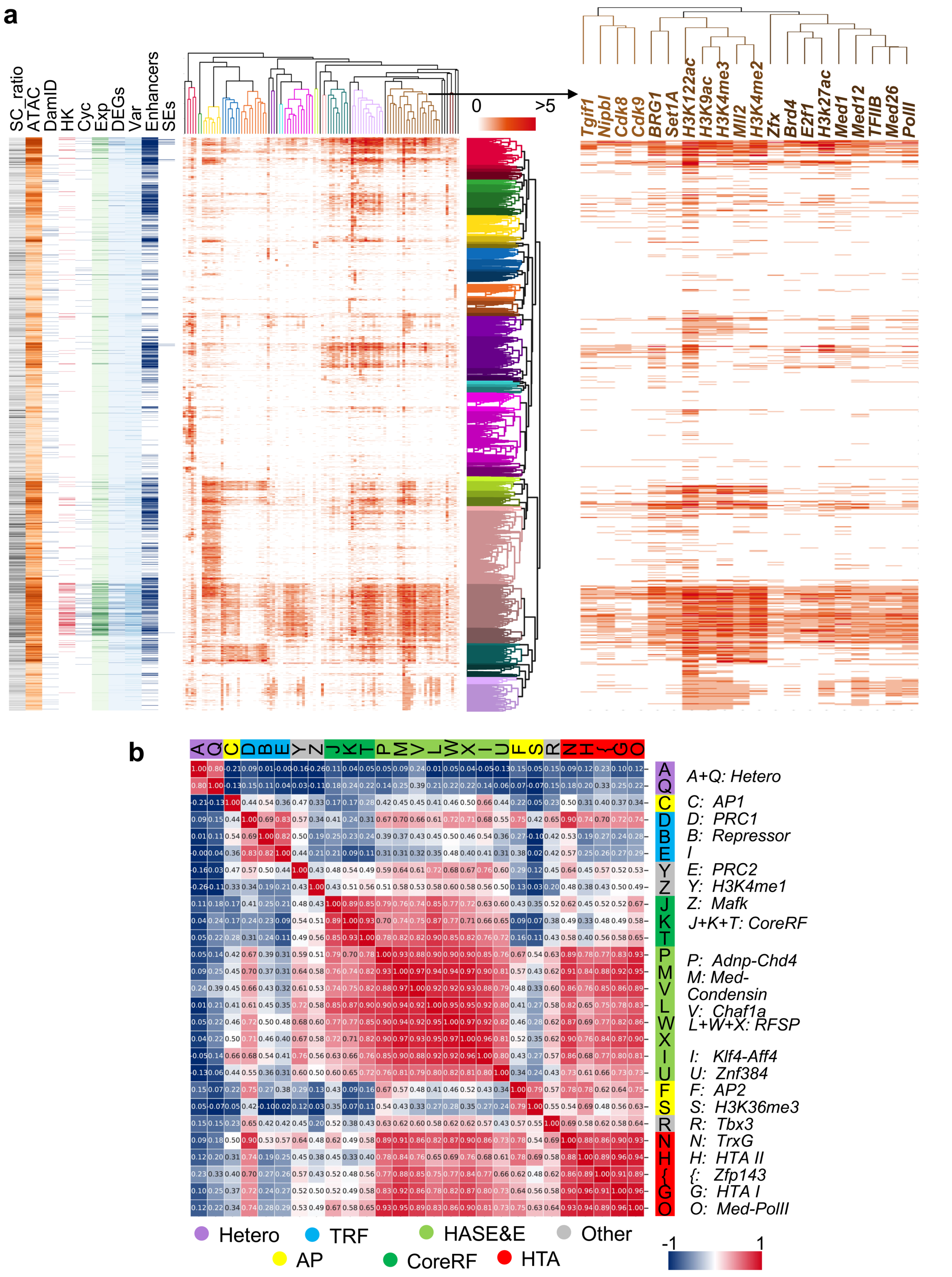

Supplementary Figure S9. a. The cluster of chromatin positions and regulatory factors. The annotation of chromatin positions by different elements or factors, including SC_ratio (the ratio of single cells forming boundaries), ATAC (ATAC-seq peaks), DamID (DamID-seq), HK (Housekeeping genes), Cyc (mark genes for cell cycle), Exp (gene expression value), DE (differential expression genes), Variable (variable scores for genes), E (enhancers), and SEs (super-enhancers) shown on the

left. Hierarchical clustering showing a further division of a certain class of regulatory factors in the right, which is shown in M-O classes of Figure 4a. **b.** The hierarchical clustering showing correlation (left) and annotation (right) of regulatory factor classes.

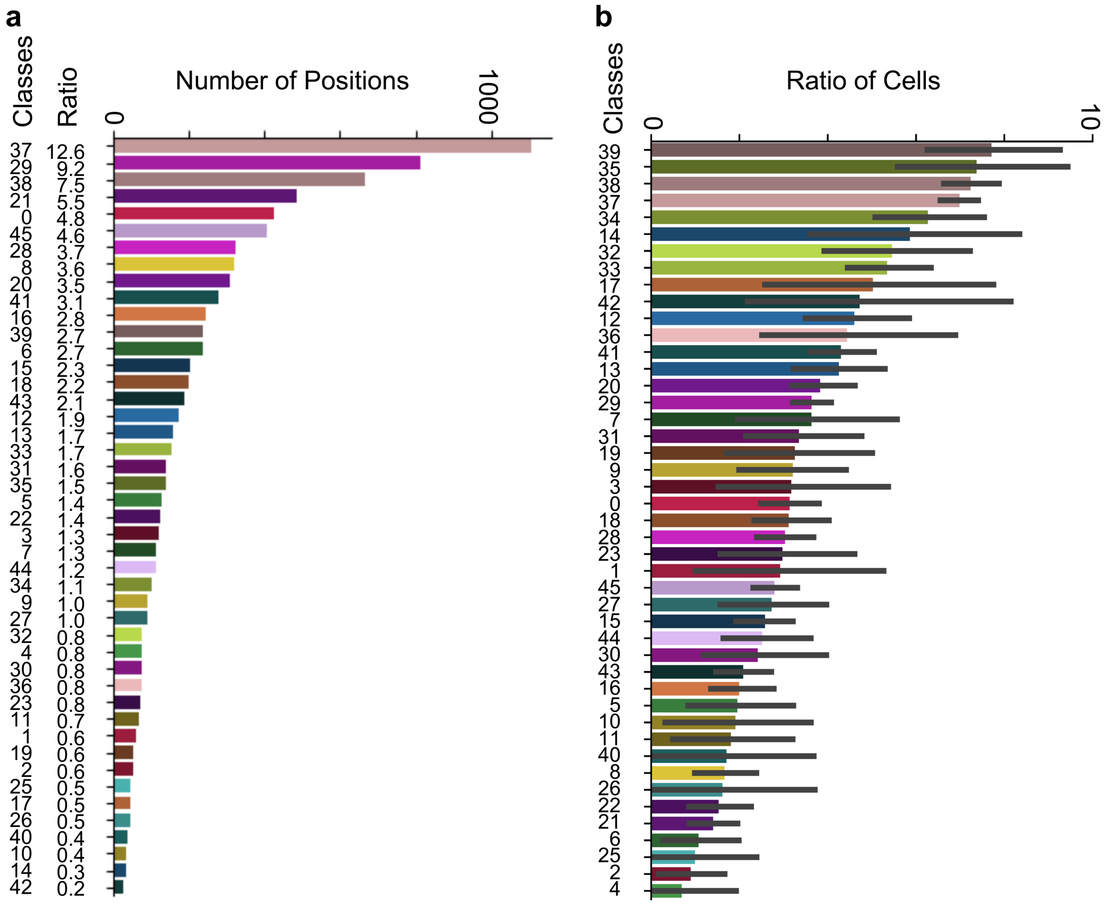

Supplementary Figure S10. a. The number and ratio of different chromatin position classes. The classes with a ratio>1% were selected for downstream analysis. **b.** The ratio distribution of single cells forming domain boundaries in different chromatin position classes.

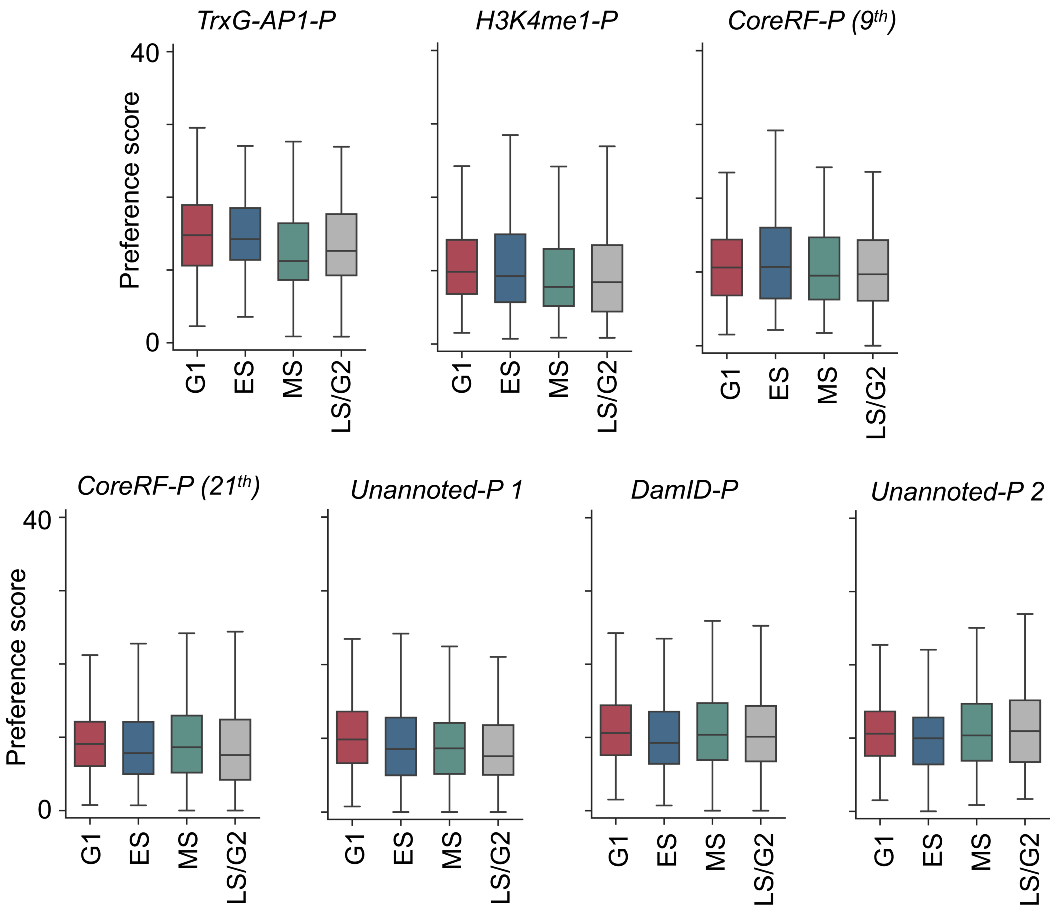

Supplementary Figure S11. Preference scores of different chromatin landscapes categories across different cell states.

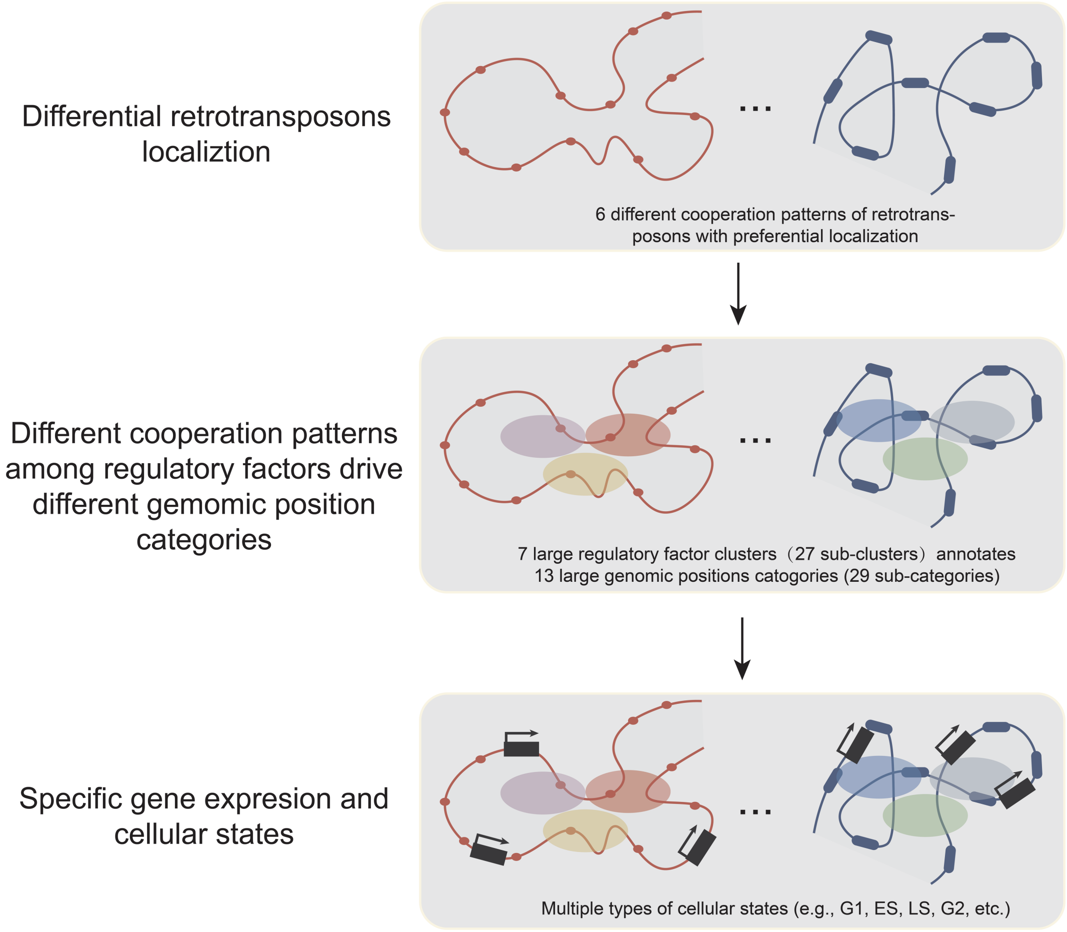

Figure S12. Different cooperation patterns of retrotransposons embedded in 3D genome architecture, regulates the formation of different chromatin states respectively, particularly the separation of compartments A/B. These different chromatin states interacting with distinct cooperative patterns of regulatory factors navigate the preference of genomic positions forming boundaries and drive the formation of higher-order chromatin structures. The process exerts gene regulatory processes and controls cell functions, even cell identity.

Supplementary Table Legends:

Supplementary Table S1. The source of regulatory factors.

Supplementary Table S2. The annotation support of regulatory factor classes.

Supplementary Table S3. The relationship among retrotransposons, genomic position categories, and the clusters of regulatory factors.

**The enrichment patterns of retrotransposons**	**Genomic position categories (sub-categories serial number)**	**The clusters of regulatory factors (typical sub-clusters or regulatory factors)**
Highly-Alu/B2-Positions	HTG-Positions (38th,39th)	➢ HTA (TrxG, HK genes);<br>➢ HASE&E (Enhancers);
	SE-Positions (0th)	➢ HASE&E (SEs,Enhancers);<br>➢ CoreRF (Sox2, Oct4, Nanog);<br>➢ HTA(TrxG, Med-PolII);
	TrxG-AP1-Positions (35th)	➢ HTA (TrxG);<br>➢ AP (CTCF, cohesin, Sa1, Sa2, Smc1, Smc3, Rad21, ZC3H11A);
Other-Alu/B2/B4-Positions	TrxG-PRC-Positions (41th, 33th)	➢ TRF (PRC1, PRC2);<br>➢ HTA (TrxG);<br>➢ AP (CTCF, cohesin, Sa1, Sa2, Smc1, Smc3, Rad21, ZC3H11A);
	H3K4me1-Positions (7th, 5th)	➢ Other (H3K4me1)

	CoreRF-Positions (9th)	➢ CoreRF (Sox2, Oct4, Nanog);<br>➢ (High chromatin accessibility);
MaLR-dominant-Positions	CoreRF-Positions (21th)	➢ CoreRF (Sox2, Oct4, Nanog);<br>➢ (DamID enrichment);
	AP1-positions (34th, 37th)	➢ AP (CTCF, cohesin, Sa1, Sa2, Smc1, Smc3, Rad21, ZC3H11A)
L1-Positions	DamID-Positions (16th, 15th, 12th, 13th)	➢ (High enrichment of DamID signal);
	AP2-Repressor-Positions (45th, 44th)	➢ AP (Yy1, H2AZ, and H3K36me3);
ERVK/L1-Positions	Hetero-Positions (29th, 28th, 20th, 31th, 27th)	➢ Hetero (Eset, H3K9me3, Sumo2, Trim28, Zfp809)
